# Controlling for Contaminants in Low-Biomass 16S rRNA Gene Sequencing Experiments

**DOI:** 10.1128/mSystems.00290-19

**Published:** 2019-06-04

**Authors:** Lisa Karstens, Mark Asquith, Sean Davin, Damien Fair, W. Thomas Gregory, Alan J. Wolfe, Jonathan Braun, Shannon McWeeney

**Affiliations:** a Division of Bioinformatics and Computational Biology, Oregon Health and Science University, Portland, Oregon, USA; b Division of Urogynecology, Oregon Health and Science University, Portland, Oregon, USA; c Division of Arthritis and Rheumatology, Oregon Health and Science University, Portland, Oregon, USA; d Department of Behavioral Neuroscience, Oregon Health and Science University, Portland, Oregon, USA; e Department of Psychiatry, Oregon Health and Science University, Portland, Oregon, USA; f Advanced Imaging Research Center, Oregon Health and Science University, Portland, Oregon, USA; g Department of Microbiology and Immunology, Stritch School of Medicine, Loyola University Chicago, Maywood, Illinois, USA; h Cedars Sinai Medical Center, Los Angeles, California, USA; University of California San Diego

**Keywords:** 16S rRNA gene sequencing, contamination, Decontam, low microbial biomass, microbiome, SourceTracker

## Abstract

Microbial communities are commonly studied using culture-independent methods, such as 16S rRNA gene sequencing. However, one challenge in accurately characterizing microbial communities is exogenous bacterial DNA contamination, particularly in low-microbial-biomass niches. Computational approaches to identify contaminant sequences have been proposed, but their performance has not been independently evaluated. To identify the impact of decreasing microbial biomass on polymicrobial 16S rRNA gene sequencing experiments, we created a mock microbial community dilution series. We evaluated four computational approaches to identify and remove contaminants, as follows: (i) filtering sequences present in a negative control, (ii) filtering sequences based on relative abundance, (iii) identifying sequences that have an inverse correlation with DNA concentration implemented in Decontam, and (iv) predicting the sequence proportion arising from defined contaminant sources implemented in SourceTracker. As expected, the proportion of contaminant bacterial DNA increased with decreasing starting microbial biomass, with 80.1% of the most diluted sample arising from contaminant sequences. Inclusion of contaminant sequences led to overinflated diversity estimates and distorted microbiome composition. All methods for contaminant identification successfully identified some contaminant sequences, which varied depending on the method parameters used and contaminant prevalence. Notably, removing sequences present in a negative control erroneously removed >20% of expected sequences. SourceTracker successfully removed over 98% of contaminants when the experimental environments were well defined. However, SourceTracker misclassified expected sequences and performed poorly when the experimental environment was unknown, failing to remove >97% of contaminants. In contrast, the Decontam frequency method did not remove expected sequences and successfully removed 70 to 90% of the contaminants.

**IMPORTANCE** The relative scarcity of microbes in low-microbial-biomass environments makes accurate determination of community composition challenging. Identifying and controlling for contaminant bacterial DNA are critical steps in understanding microbial communities from these low-biomass environments. Our study introduces the use of a mock community dilution series as a positive control and evaluates four computational strategies that can identify contaminants in 16S rRNA gene sequencing experiments in order to remove them from downstream analyses. The appropriate computational approach for removing contaminant sequences from an experiment depends on prior knowledge about the microbial environment under investigation and can be evaluated with a dilution series of a mock microbial community.

## Abstract (precis)

The relative scarcity of microbes in low-microbial-biomass environments makes accurate determination of community composition challenging. Identifying and controlling for contaminant bacterial DNA are critical steps in understanding microbial communities from these low-biomass environments. Our study introduces the use of a mock community dilution series as a positive control and evaluates four computational strategies that can identify contaminants in 16S rRNA gene sequencing experiments in order to remove them from downstream analyses. The appropriate computational approach for removing contaminant sequences from an experiment depends on prior knowledge about the microbial environment under investigation and can be evaluated with a dilution series of a mock microbial community.

## INTRODUCTION

Advances in genomic sequencing have transformed our ability to identify and study microbes without depending on culture-based laboratory techniques. A common method used to study microbial communities is sequencing of marker genes, such as the 16S rRNA gene. In this method, the bacterial DNA is extracted from a sample, amplified by PCR, and then sequenced. This technique is relatively inexpensive and easy to perform and has increased our ability to detect and identify microbes in a variety of environments, including those of low microbial biomass, such as the upper atmosphere (1), lower airway (2), and urinary bladder (3–5).

The unexpected discovery of microbes in these niches is exciting and can revolutionize our understanding of these environments. However, a major challenge hindering our ability to accurately characterize microbial communities in these environments is bacterial DNA contamination from exogenous sources that are introduced during sample collection and processing. Contaminant bacteria introduced to the sample prior to PCR can dominate the composition of low-microbial-biomass samples (6–9), comprising over 80% of the sample in extreme cases (10). Failure to account for contaminants may also affect biological conclusions drawn from studies, such as inflating alpha-diversity metrics, distorting the abundance of true microbial members of the environment (10), and altering differences between clinical groups (7, 9, 11).

Currently, there is no standard method to minimize or control for contaminants in 16S rRNA gene sequencing experiments. During sample processing, procedures can be taken to minimize the amount of exogenous DNA introduced into the samples. These include pretreating reagents in an attempt to remove exogenous DNA (10, 12) and using DNA extraction kits designed specifically to minimize contamination. However, techniques involving pretreating reagents are challenging and may be ineffective when examining low-microbial-biomass samples (10).

Several approaches to objectively remove contaminants after 16S rRNA gene sequencing have been suggested. One directly identifies and removes sequences that have been previously identified as contaminants in published databases or reference lists (7). However, this is a generalized approach and might not accurately reflect the contamination present in the actual experiment. A second approach applies an abundance filter to remove all sequences that are below a defined relative abundance threshold (13, 14). However, this method assumes that all microbial contaminants have a low relative abundance, which may not be the case particularly for low-microbial-biomass samples. This also will remove all noncontaminant sequences below this threshold. A third approach is to remove sequences that are present in a negative-control sample (15, 16). However, this approach may be too harsh since low levels of real sequences from the sequence run may be present in the negative control due to multiplexing artifacts (17, 18). This leads to removing bacterial sequences that are actually biologically relevant; thus, this approach has been found to be too strict (10). A fourth approach is to identify bacterial sequences that have an inverse correlation with bacterial DNA concentration after 16S rRNA library preparation (9, 13, 19). This has been recently implemented in an open-source R package, Decontam (20). A fifth approach uses a Bayesian approach implemented in SourceTracker (21) to predict the proportion of an experimental sample that arose from a defined contaminant source.

While the above-described methods have been proposed to identify and remove contaminants from 16S rRNA gene sequencing studies, there is no current guidance for evaluating their success. Here, we propose using a mock microbial community dilution series to evaluate the effectiveness of approaches to minimize and remove contaminants. The use of mock microbial communities as a positive control for 16S rRNA gene sequencing experiments has been advocated (22), though it is unclear how often they are used in practice. Mock microbial communities are composed of mixtures of known bacterial composition and are subject to the same experimental and computational processing as experimental samples. Since the expected composition of the mock microbial community is known, it can be used to identify problems in the experimental protocol, understand biases introduced in the experimental protocol (i.e., PCR amplification bias), optimize the bioinformatics workflow used, or develop new methods.

In this study, we use a dilution series of a mock microbial community to determine the success of existing approaches to control for laboratory contaminants in studies involving low-microbial-biomass samples. We demonstrate that a dilution series of a mock microbial community is a valuable tool to evaluate optimal inputs and parameters for two filtering approaches and two computational approaches that have been proposed for contaminant identification from low-microbial-biomass samples.

## RESULTS

To identify the impact of exogenous bacterial DNA on low-microbial-biomass microbiome studies, we used 16S rRNA gene sequencing of a mock microbial community that had undergone eight rounds of serial 3-fold dilutions (see Table S1 in the supplemental material). This allowed us to mimic the decreasing biomass of biological samples and its effect on the fidelity of 16S rRNA sequencing. We chose a mock microbial community since most microbiome studies aim to identify mixtures of bacteria rather than pure bacterial isolates. In mock communities, the expected 16S rRNA gene sequences are known; thus, any unexpected sequences identified in the analysis of the dilution series can be attributed to error. These errors can arise from sequencer errors (base miscalls or barcode cross talk), chimeric sequences, or laboratory contaminants.

10.1128/mSystems.00290-19.3 TABLE S1 Estimated number of bacterial cells that yielded the DNA used in 16S rRNA gene PCR reaction for the mock microbial dilution series and DNA concentration measured by nanodrop. Download Table S1, CSV file, 0.00 MB. Copyright © 2019 Karstens et al. 2019 Karstens et al. This content is distributed under the terms of the Creative Commons Attribution 4.0 International license.

### 16S rRNA gene sequencing of the mock microbial dilution series and negative control.

**FIG 1:**
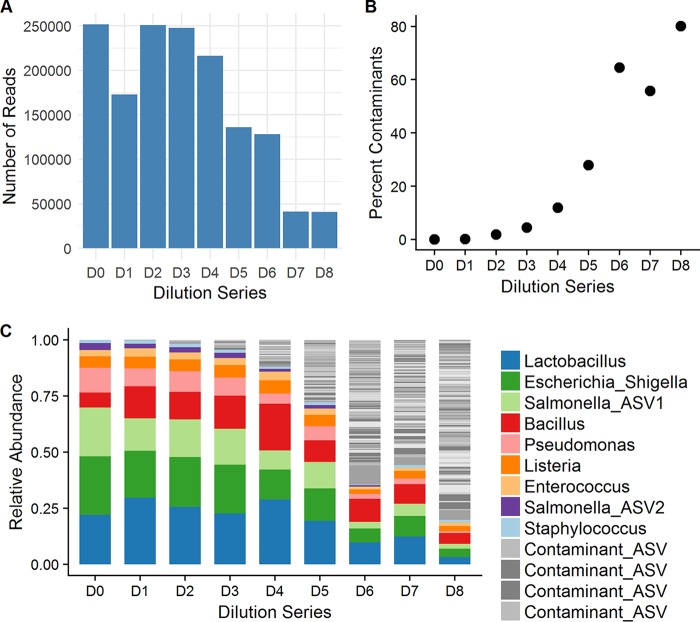
Analysis of a mock microbial community dilution series reveals that contaminating bacteria increase with decreasing starting DNA. A mock microbial community consisting of 8 known bacteria was subject to 8 series of a 3-fold dilution (1:3 through 1:6,561), subject to bacterial DNA isolation and amplification, and sequenced with the Illumina MiSeq platform. (A) Number of reads per sample. (B) The proportion of reads from contaminant DNA increased with the amount of dilution. (C) Stacked bar plot representing the bacteria identified in each sample. The expected ASVs from the mock microbial community are displayed in color, while all other bacterial ASVs are in grayscale.

The total number of paired-end reads from the mock community dilution samples was 2,555,160. Following quality filtering, a total of 1,675,028 sequences were further analyzed and grouped into 1,414 amplicon sequence variants (ASVs). Each sample in the mock community dilution series had between 40,927 and 251,419 reads. As expected, the number of reads per sample generally decreased with increased dilution, though not linearly (Fig. 1A and Table 1).

**TABLE 1:** Impact of decreasing starting material for 16S rRNA gene sequencing

Parameter	Data by dilution								
	D0	D1	D2	D3	D4	D5	D6	D7	D8
No. of reads	251,419	172,915	250,861	247,581	216,341	136,081	128,053	41,071	40,927
No. of unique ASVs	18	20	114	172	262	312	381	147	193
No. of unique genera	15	16	64	61	80	86	107	58	74
% contaminants^*a*^	0.1	0.1	1.8	4.5	12.0	27.9	64.5	55.8	80.1

a Percent contaminants calculated as the percentage of sequences in each sample that were not an exact match to the mock microbial community reference sequences.

**(i) Undiluted mock community sample composition.** The mock microbial community consisted of 8 bacterial species from 8 distinct genera (see Materials and Methods). These mapped to 9 unique expected mock community ASVs since sequences from one expected constituent, Salmonella enterica, were split into two ASVs based on a single nucleotide variation (G-to-T transition). In total, sequencing of the undiluted mock community resulted in 18 unique ASVs, which corresponded to 15 known genera (Table 1). The expected mock community ASVs were represented in 99.95% of the sample. The other 9 unexpected ASVs totaled 0.05% of the sequences. Due to their low relative abundance and lack of other explanation, these were likely either contaminants from sample processing or barcode cross talk from sequencing.

**(ii) Negative-control composition.** The negative control was composed of 655 ASVs that mapped to 136 genera. The most abundant taxa were the genus Bacteroides (15.1%), followed by the family *Lachnospiraceae* (6.9%) and the genera Faecalibacterium (6.3%) and Ruminiclostridium_6 (6.0%). Within the sample, 21 genera were present at relative abundances between 1% and 5%, whereas the remaining genera were present at relative abundances of less than 1%. Three of the 8 mock community ASVs were present in the negative control. These were in low abundance (0.03% to 1.1%), making up 1.7% of the total relative abundance of the negative-control sample.

**(iii) Mock community dilution series composition.** The amount of contaminant DNA increased with subsequent dilutions of the mock microbial dilution series, indicated by an increased number of ASVs and bacterial genera (Table 1) that were not in the mock microbial community. As expected, the numbers of ASVs and genera increased with decreased starting microbial biomass for dilutions D1 to D6. However, this trend did not hold for the most diluted samples (D7 and D8, Table 1). This is likely due to the lower number of total sequences from these samples. We did identify an increased percentage of contaminant ASVs, which had a relationship with dilution (Fig. 1B).

There were 937 contaminant ASVs in the dilution series. After two rounds of dilution, contaminant ASVs became more predominant in the microbial community profiles (Fig. 1B and C), with contaminants having relative abundances greater than 0.5%. After the 6th round of dilution (D6), contaminants contributed over 50% to the estimated community composition. Importantly, contaminant genera in these dilutions were detected with relative abundances greater than 10%, making individual contaminants easily mistaken for signal from actual microbes present in a sample.

The contaminants identified with abundances greater than 1% in a sample are listed in Table S2. The most prevalent ASV was the genus Bacteroides, present at relative abundances of up to 8.6% in the most diluted samples (D6 to D8), followed by two ASVs identified as the *Lachnospiraceae NK4A136* group with relative abundances of up to 3.2%. Other contaminant ASVs comprised less than 2% relative abundance per sample.

10.1128/mSystems.00290-19.4 TABLE S2 Taxonomic classification of contaminant ASVs with at least 1% abundance in at least 1 sample. Download Table S2, CSV file, 0.01 MB. Copyright © 2019 Karstens et al. 2019 Karstens et al. This content is distributed under the terms of the Creative Commons Attribution 4.0 International license.

One hundred ninety-four of the 937 contaminant ASVs were present in the negative-control sample. ASVs from the negative control made up 35.2% to 62.7% of the contaminant sequences per sample (average, 50.1%). Of the ASVs that were not present in the negative control, 675 ASVs were only present in a single dilution series sample, with 2 to 696 sequences per sample (mean, 77.8 sequences). These ASVs were present in other samples multiplexed on the same sequencing run and are likely due to barcode cross talk.

**FIG 2:**
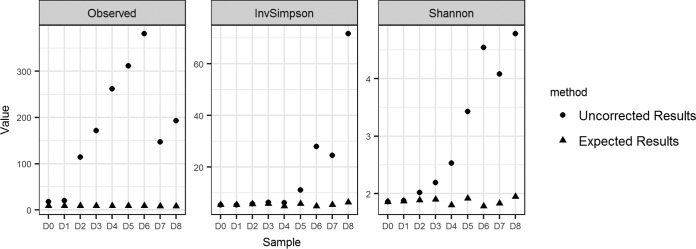
Impact of contaminants on common alpha-diversity measures. Failure to remove contaminants from the data set leads to increased estimates of alpha diversity evaluated by the number of observed ASVs (Observed) inverse Simpson index (InvSimpson), and Shannon diversity index (Shannon). Expected results were calculated based on the expected mock community ASVs in each dilution sample.

**(iv) Impact of contaminants on alpha-diversity metrics.** To identify the impact of including contaminants in downstream microbiome analyses, we calculated the following commonly used measures of alpha diversity: observed number of ASVs, the inverse Simpson index, and the Shannon index. The expected values for these measures based on the expected mock microbial community sequences are 9, 1.86, and 5.40, respectively. The inclusion of contaminant sequences led to increases in all estimates (observed ASVs, 18 to 381; inverse Simpson index, 5.41 to 71.58; Shannon index, 1.86 to 4.78; see Fig. 2).

### Methods to identify and remove contaminants.

We evaluated the following four approaches to identify contaminant ASVs from the mock microbial dilution series: filtering ASVs present in the negative-control sample, filtering based on ASV relative abundance, using the frequency method in Decontam, and using the predictive modeling approach in SourceTracker (Table 2).

**TABLE 2:** Details of computational methods for identifying and removing contaminants

Method	Details	Parameters evaluated
Filter, negative control	ASVs present in the negative control are removed	None
Filter, abundance	ASVs below a relative abundance threshold are removed	Abundance threshold, 0.01%, 0.10%, or 1.00%
Decontam, frequency	ASVs with a correlation with DNA concentration are removed	Threshold parameter, 0.1, 0.2, 0.3, 0.4, or 0.5
SourceTracker, scenario 1; experimental source environment is well defined	Proportions of ASVs predicted to not be from a defined experimental source are removed	Source environments; for case 1, mock community profile + contaminant profile + negative-control profile; for case 2, mock community profile + negative-control profile
SourceTracker, scenario 2; experimental source environment is not defined	Proportion of ASVs predicted to be from a contaminant source are removed	Source environments; for case 1, contaminant profile + negative-control profile; for case 2, negative-control profile

**(i) Contaminant removal by filtering.** We evaluated using a negative control or relative abundance threshold for identifying contaminant ASVs. For filtering by negative control, we removed any ASV with a nonzero abundance in the negative control from the mock community dilution series. Relative abundance filtering was performed by setting a filter to remove ASVs with an abundance less than a specified relative abundance (0.01, 0.1, or 1.0) from each sample.

**(ii) Contaminant removal by correlation with DNA concentration using Decontam.** The frequency method in the open-source R package Decontam (20) identifies an inverse relationship between sample amplicon concentration after library preparation and individual ASV abundance. This relationship has been reported for both Illumina sequencing (9) and pyrosequencing (13) and provides an objective way to identify contaminants. We evaluated the performance of the Decontam frequency method using a variety of user-defined thresholds, ranging from the default 0.1 to 0.5. These thresholds dictate the value at which an ASV is classified as a contaminant or noncontaminant.

**(iii) Contaminant removal with SourceTracker.** We used the SourceTracker algorithm (21) to identify and remove contaminant ASVs from the mock microbial dilution series. SourceTracker uses a Bayesian approach to predict the proportion of each ASV in an experimental sample that arises from defined “source” environments that the user provides. The source environments can be created from existing sequence databases (e.g., of published laboratory contaminants) or by using samples that originate from a known potential source (e.g., experimental blank controls). For each ASV, the probability of the ASV arising from each defined source is calculated. If an ASV does not fit any of the defined sources, it is classified as arising from an unknown source.

We tested two scenarios for recovering the expected mock microbial community profiles from the mock microbial dilution series using SourceTracker. In the first scenario, we created a source environment from the expected sequences present in the undiluted mock microbial community sample, mimicking the scenario when the experimental environment is well defined. In the second scenario, the expected mock microbial community is unknown; the proportion of sequences not predicted to be from the defined negative-control source or contaminant profile source is considered the contamination-corrected profile. The second scenario is the more commonly encountered scenario, where the microbial environment that is being studied is poorly defined. For each scenario, we evaluated two cases defining different contaminant source environments. In case 1, the contaminant source environments were defined as both a contaminant profile and a negative-control profile. In case 2, we used only the negative control as a contaminant source environment to test if the negative controls alone were enough to identify contaminants.

### Evaluation of methods to remove contaminants.

To evaluate the overall performance of each contaminant removal method, ASVs were classified as being correctly or incorrectly identified as a contaminant ASV or a mock community ASV. A contaminant ASV is an ASV that was not expected to be part of the mock community. A mock community ASV is an ASV that is expected to occur in the mock community. We calculated the overall accuracy of each method, i.e., the ability to differentiate between contaminant ASVs and mock community ASVs. To evaluate the success of contaminant removal methods, we calculated commonly used alpha-diversity metrics and the relative abundance of the corrected microbial profiles after contaminant removal.

**FIG 3:**
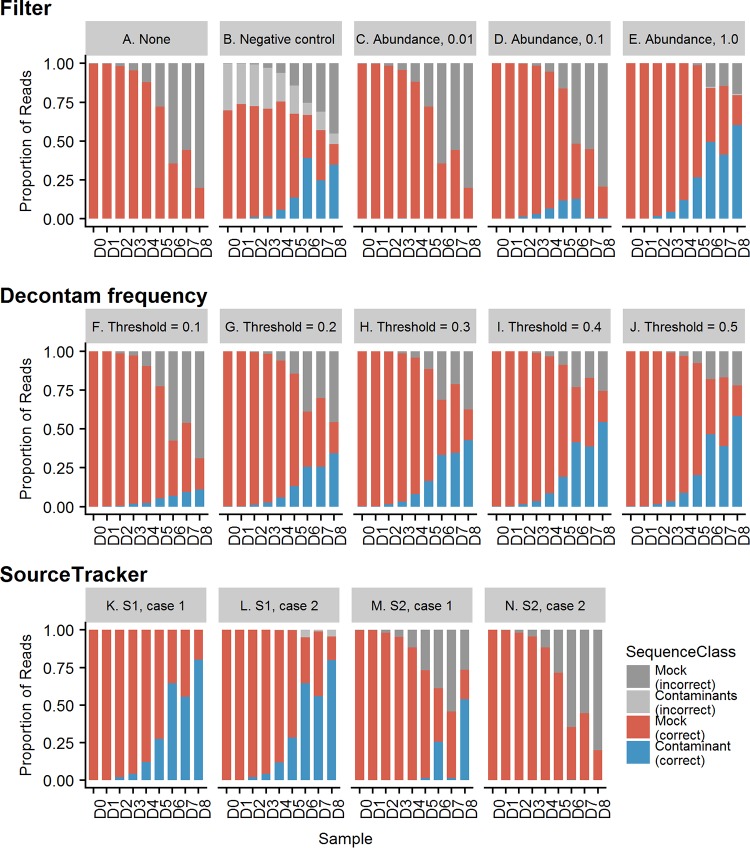
Classification of ASVs. Red, mock community ASVs correctly classified; light gray, mock community ASVs incorrectly classified; blue, contaminant ASVs correctly classified; dark gray, contaminant ASVs incorrectly classified. (A) Not correcting for contaminants leads to a large proportion of sequences being incorrectly considered mock ASVs across the dilution series. (B) Filtering by removing ASVs present in the negative control incorrectly classified many mock community ASVs as contaminants and misclassified many contaminant ASVs as mock community ASVs in the diluted samples. (C to E) Abundance filtering required an abundance of 1% to classify the majority of contaminant ASVs correctly, but it also removed mock community ASVs (E). (F to J) The Decontam frequency method did not misclassify any mock community ASV sequences and correctly classified the majority of contaminant ASVs. In highly diluted samples (D6 to D8), contaminant ASVs are misclassified as mock community ASVs. (K to N) SourceTracker performs well, correctly classifying the majority of mock community and contaminant ASVs, though some mock community ASVs are classified incorrectly as contaminant ASVs (K and L). However, in the scenario where the experimental environment is not well defined (M and N), many of the ASVs are incorrectly classified as mock community ASVs. S1, scenario 1; S2, scenario 2.

**(i) Classification of ASVs.** To evaluate the overall performance of each contaminant removal method, ASVs were classified as being correctly or incorrectly identified as mock community or contaminants (Fig. 3 and Table S3). Using the negative control to remove contaminant ASVs misclassified mock community ASVs as contaminant ASVs (20.1 to 33.9% of mock community ASVs; Fig. 3A and Table S3) and only classified 33.5 to 62.7% of the contaminants correctly. Contaminant removal using a low relative abundance filter (0.01%) misclassified the majority of contaminant ASVs (Fig. 3B and Table S3). An abundance filter of 0.1% successfully classified the contaminant ASVs when the prevalence of contaminants was low, but it misclassified the majority of contaminant ASVs (>50%) when the prevalence of contaminants increased (Fig. 3C, samples D5 to D8, and Table S3). An abundance filter of 1% successfully classified greater than 70% of the contaminant ASVs correctly across the dilution series but misclassified up to 3.9% of the mock community ASVs in the most diluted samples (Fig. 3D, D6 to D8, and Table S3). The Decontam frequency method correctly classified all mock community ASVs and correctly classified 10.9% to 90.4% of the contaminant ASVs (Fig. 3E to I and Table S3). As expected, increasing the threshold led to an increased proportion of ASVs being correctly classified as contaminants across the dilution series, with a threshold of 0.5 correctly classifying over 69.6% of the contaminant ASVs correctly across the dilution series. SourceTracker correctly classified the majority of mock community ASVs and contaminant ASVs in the scenario with well-defined experimental environments, with less than 0.2% of the contaminant ASVs misclassified. However, SourceTracker also misclassified mock community ASVs (0 to 21.7%) (Fig. 3J and K and Table S3). In the experimental scenario where the expected source environment was not well defined, SourceTracker failed to correctly classify many contaminant ASVs (0 to 67.0% across the dilution series; Fig. 3L and M and Table S3).

10.1128/mSystems.00290-19.5 TABLE S3 Percentage of contaminant ASVs and mock community ASVs removed using each contaminant removal method. Download Table S3, XLSX file, 0.03 MB. Copyright © 2019 Karstens et al. 2019 Karstens et al. This content is distributed under the terms of the Creative Commons Attribution 4.0 International license.

**FIG 4:**
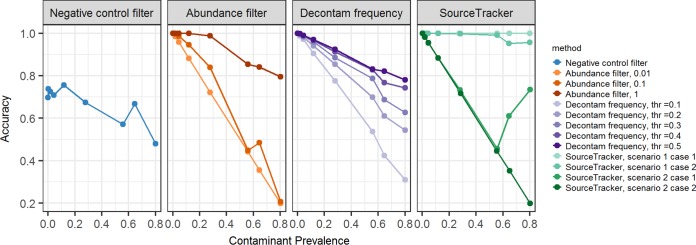
Accuracy of methods to identify contaminants. The negative-control filter has poor accuracy, regardless of prevalence of contaminants. All other methods have high accuracy when the prevalence of contaminants is <5%. As the prevalence of contaminants increases, the accuracy of most methods drops, with the exception of SourceTracker with well-defined experimental conditions. Thr, threshold.

The overall accuracy of each method is presented in Fig. 4. All methods except the negative-control filter had high accuracy (>0.95) when the prevalence of contaminant in the sample was low (<5% of the total sample composition). For moderate levels of contaminants, the accuracy dropped below 0.90 for most methods but remained above 0.90 for the 1% abundance filter, the Decontam frequency method with a threshold above 0.3, and SourceTracker with well-defined experimental environments. For high levels of contaminants (>50% of the sample composition), accuracy decreased for all methods but remained above 0.90 for SourceTracker with well-defined experimental environments and above 0.75 for the Decontam frequency method with a threshold of 0.5 and an abundance filter of 1%.

### Results after contaminant removal.

**FIG 5:**
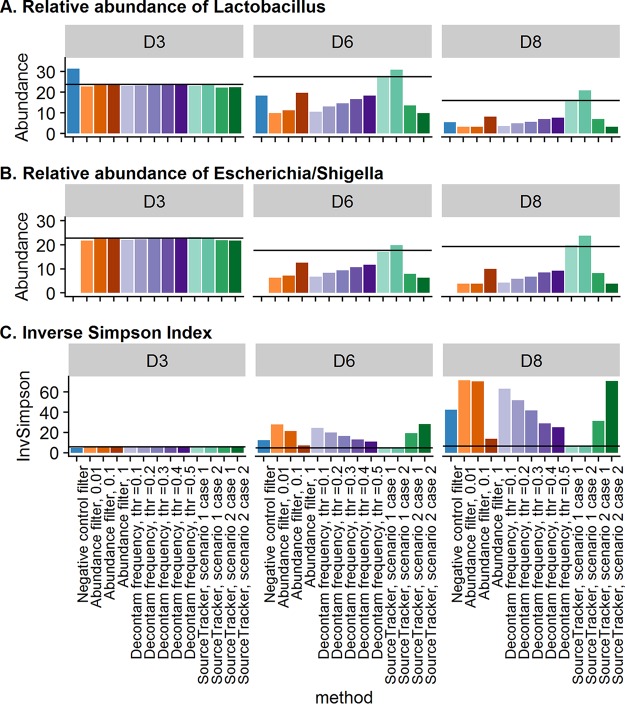
Example of the effect of contaminant removal on common microbiome summary measures (relative abundance of ASVs, alpha diversity). (A) Relative abundance of *Lactobacillus*. (B) Relative abundance of *Escherichia/Shigella*. (C) Alpha diversity summarized by the inverse Simpson index. For brevity, results are shown for three dilution series samples representing low (less than 5%, D3), moderate (between 10% and 50%; D6), and high (greater than 50%, D8) levels of contaminant ASVs. Expected results shown by solid black line are based on the composition of the expected mock community ASVs in the specified dilution sample. See Fig. S1 and S2 for results across the entire dilution series.

To demonstrate the ability of contaminant removal methods to lead to a more accurate representation of microbial profiles, we calculated commonly used alpha-diversity metrics and the relative abundance of the corrected microbial profiles (Fig. 5). For brevity, the results are displayed for three dilution series samples each with either low, moderate, or high levels of initial contaminants. Overall, contaminant removal methods worked best on samples with low to moderate levels of contaminant. Once the prevalence of contaminants increased to above 50%, most methods were unable to accurately identify contaminants. Using the negative-control filter distorted the relative abundances of expected mock community ASVs. For example, Lactobacillus spp. were overrepresented in samples with low levels of contaminants (i.e., D3, Fig. 5A), and Escherichia/Shigella spp. were removed from the data set since they were present in the negative-control sample (Fig. 5B). The methods best able to recover the expected microbial community composition were SourceTracker, with well-defined experimental environments, followed by the Decontam frequency method, with a threshold of 0.5 and an abundance filter of 1.0%.

10.1128/mSystems.00290-19.1 FIG S1 Recovered microbial community profiles after contaminant removal. Download FIG S1, PDF file, 0.02 MB. Copyright © 2019 Karstens et al. 2019 Karstens et al. This content is distributed under the terms of the Creative Commons Attribution 4.0 International license.

10.1128/mSystems.00290-19.2 FIG S2 Alpha-diversity metrics after contaminant removal for all methods. Download FIG S2, PDF file, 0.02 MB. Copyright © 2019 Karstens et al. 2019 Karstens et al. This content is distributed under the terms of the Creative Commons Attribution 4.0 International license.

## DISCUSSION

### Impact of contaminants on microbial community composition.

Our study demonstrates the impact of laboratory contaminants on 16S rRNA gene sequencing experiments of various starting microbial biomass levels on a mixed microbial community. The diluted mock microbial community samples showed a marked increase in the number of ASVs and, thus, genera with decreasing starting material. This led to increased estimates of commonly used alpha-diversity metrics incorporating species or genus richness. Highly diluted samples also demonstrated contaminant bacteria that were higher in abundance than the expected sequences. The inclusion of such large amounts of contaminant taxa can artificially decrease the relative abundance of the actual expected microbial communities. Thus, our findings further extend the work of Salter et al. (7), demonstrating the magnitude of error from biological data sets harboring contaminants unaddressed by suitable control procedures on a mixed microbial sample.

Contaminated laboratory reagents in 16S rRNA gene-based experiments have long been recognized in the scientific literature (23). Several groups have recently investigated the impact of bacteria arising from DNA extraction kits and other commonly used laboratory reagents specifically on 16S rRNA gene sequencing experiments. Salter et al. performed a serial dilution of a pure bacterial isolate to assess contaminants (7). Similar to our study, after 4 10-fold dilutions of a single bacterial isolate, they found that samples were predominantly composed of contaminant DNA sequences. Glassing et al. demonstrated the effect of contaminants on biological samples of low microbial biomass and evaluated methods for mitigating contaminant contributions using a variety of negative controls (10). These studies exemplify the need for researchers to be adamant about the contaminant control procedures used.

### Identifying contaminants in low-microbial-biomass experiments.

Using a dilution series of mock microbial communities, where the composition and sequences of the mock microbial community are known, we evaluated several strategies for identifying and removing contaminant sequences. In our evaluation, we used a negative control and an abundance filter, as well as two available computational methods to minimize and remove contaminant sequences from downstream analyses. To our knowledge, this is the first study examining the performance of these methods.

Our data suggest that the use of negative controls alone is insufficient to inform researchers of appropriate measures to minimize contaminants from their experiments. While we found that ASVs present in the negative-control sample were also present in the mock community dilutions, we also identified many contaminant ASVs in the mock microbial dilution samples that were absent from the negative control. Furthermore, we found that three of the nine expected sequences from the mock microbial community were also present in the negative control. This could be due to sample cross-contamination, the presence of closely related organisms, or barcode cross talk (18). Removing all sequences present in the negative control led to erroneous removal of many of the expected mock community dilution sequences.

We also evaluated the use of a relative abundance filter to remove contaminant ASVs, with mixed success. We evaluated a range of relative abundances and found low-abundance filters (0.01% and 0.1%) to be too conservative, leaving the majority of contaminant ASVs in the data set, especially as the prevalence of contaminants increased. The use of a higher relative abundance (1%) removed a large number of contaminant ASVs but also removed mock community ASVs and missed contaminant ASVs present at abundances greater than 1%, particularly in the most diluted samples (D7 and D8). Thus, an appropriate filter that balances contaminant removal with retention of real ASVs is challenging.

In addition to filtering methods, computational approaches have been developed to identify and remove potential contaminants. These methods include identifying correlations with ASV or operational taxonomic unit (OTU) frequency and starting DNA material measured either before library preparation with a PicoGreen assay or a NanoDrop spectrophotometer (9) or by more accurate but more labor-intensive quantitative PCR (qPCR) (19). We evaluated this approach as implemented in the Decontam package available in R (20). The Decontam frequency method demonstrated reasonable performance by removing the majority of contaminants while not removing any expected mock community ASVs with all tested thresholds, though some contaminants still remained in the most diluted samples. At the highest threshold evaluated (0.5), the Decontam frequency method removed 70 to 90% of contaminant ASVs. The remaining contaminant ASVs were in low abundance, and additional filtering steps, such as low-abundance filtering or the prevalence method available in Decontam, would likely further remove these contaminant ASVs.

Another approach uses Bayesian modeling to estimate the proportion of contaminants as implemented in SourceTracker. Our study indicates that using SourceTracker is an excellent approach if the environment under investigation is well defined, removing 98% of contaminants. This scenario represents a highly controlled experiment or extremely well-characterized environment where the microbial composition is well defined. However, SourceTracker performed poorly in the scenario where the environment under investigation is unknown, which is likely the case for most low-microbial-biomass environments. In this scenario, SourceTracker identified less than 1% of contaminants.

Another consideration for selecting a contaminant removal method is the format of the results, which may limit downstream analysis. The filtering approaches and Decontam classify individual ASVs (or operational taxonomic units [OTUs]) as either being contaminants or not, and ASVs/OTUs that are identified as contaminants are removed from the entire data set. This has the benefit of retaining the raw read count of the data, but it does not permit removal of a proportion of a contaminating sequence, which may be desirable if a known contaminant is also known to be part of the experimental environment being studied. In contrast, SourceTracker has the benefit of estimating proportions of sequences that arise from a contaminant source rather than removing the entire sequence from the data set. However, with the current implementation, it is unclear how to recover raw sequencing counts from the results. Furthermore, SourceTracker takes a significant time to run, which increases with increasing sequencing depth. To overcome this, it is common/recommended practice to subsample the data prior to using SourceTracker, which is currently a controversial practice in microbiome analysis (24).

### Limitations.

Our study used a commercial mock microbial community consisting of 8 known bacterial species; it also included 2 fungal species (which do not possess a 16S rRNA gene and thus were not analyzed in this study). Mock microbial communities that are representative of or have characteristics similar to the expected experimental microbial community can also be generated and may provide a more robust estimate of the experimental contaminants present in a study. Furthermore, in our study, all negative controls were barcoded with the same barcode, limiting their utility. For example, we were unable to use the prevalence method for contaminant removal that is available in the Decontam package. We also limited our evaluation to computational strategies for identifying contaminants that do not require additional experimental information, such as spike-in controls (25), KatharoSeq (26), or qPCR (19).

### Conclusion.

Controlling for contaminants in low-microbial-biomass experiments remains an important and unsolved problem, particularly in the age of easily accessible high-throughput next-generation sequencing. We demonstrate that using a dilution series of a mock microbial community can provide researchers studying low-microbial-biomass environments with an effective means to evaluate laboratory contaminants in 16S rRNA gene sequencing experiments. This includes identifying the actual background noise specific for a particular experiment, as well as for evaluating the success and optimizing the parameters of methods used to identify exogenous contaminant DNA from experiments. Since the exogenous DNA present in DNA extraction kits has been shown to vary across kit types and different lots of the same kit, a control such as a mock community dilution series is an important addition to each 16S rRNA gene sequencing experiment in order to identify experiment-specific contaminants. This is particularly important for studies investigating environments with low microbial biomass, such as urine, lower airway, and upper atmosphere. We recommend that investigators use a whole-cell mock community dilution series consisting of at least high, medium, and low dilutions representing the expected range of DNA concentration in the environment being studied in addition to extraction blanks as negative controls.

We demonstrated that identifying contaminants in 16S rRNA gene sequencing experiments is challenging, and that computational strategies can be used to identify and remove contaminant ASVs. We found that aside from the negative-control filter, each method had conditions under which it performed well. Most methods did not perform well once the amount of contaminants increased to one-third of the sample. We found that the Decontam frequency method was robust, did not misclassify any mock community ASVs, and did not require prior information about the microbial community being studied.

Importantly, it is likely that a single approach for removing contaminants from low-biomass samples will not work equivalently across all sequencing studies investigating low-microbial-biomass environments. Thus, it is important that the method used for controlling contaminants be evaluated and reported upon publication. A mock microbial dilution series can serve as a way to evaluate different methods of contaminant removal for a specific experiment. This will ensure that the science is transparent and will make results more robust and reproducible.

## MATERIALS AND METHODS

### Microbial mock community dilution.

ZymoBIOMIC mock community standards were used (Zymo Research). This mock community consisted of 8 bacterial species (Pseudomonas aeruginosa, Escherichia coli, Salmonella enterica, Lactobacillus fermentum, Enterococcus faecalis, Staphylococcus aureus, Listeria monocytogenes, and Bacillus subtilis) and two fungal species (Saccharomyces cerevisiae and Cryptococcus neoformans). The mock community was diluted with microbial DNA-free water (Qiagen) in 8 rounds of a serial 3-fold dilution prior to DNA extraction. A total of 50 μl of community standard (equivalent to ∼1.5 × 10^9^ total bacteria) was used as the highest microbial standard for DNA extraction (see Table S1). Blank controls of microbial DNA-free water also were used and subjected to all processing steps.

### DNA extraction and PCR amplification.

A total of 50 μl of each serially diluted microbial standard was subject to DNA extraction using the cultured cell protocol supplied with the DNeasy blood and tissue kit (Qiagen, Germany), as per the manufacturer’s instructions. DNA was eluted in a total volume of 50 μl. The extracted DNA was quantified and quality checked at *A*_260_/*A*_280_ (NanoDrop spectrophotometer; Thermo Fisher Scientific, USA) prior to amplification by PCR. Bacterial DNA was amplified by PCR using Golay barcoded primers, which target the V4 region of 16S rRNA genes (37). Template DNA was amplified in triplicate using the GoTaq Hot Start polymerase kit (Promega, USA). One microliter of template DNA and 1 μl of a unique barcoded reverse primer were added to 48 μl of master mix containing 1× colorless reaction buffer, 1.5 mM MgCl_2_, 0.2 mM dinucleoside triphosphates (dNTPs), 0.2 mM forward primer, and 1.25 U of polymerase enzyme. The reaction volumes were placed in a thermocycler and run through the following conditions: 94°C for 3 min (initial denaturation), followed by 35 cycles of 94°C for 45 s (denaturation), 55°C for 40 s (annealing), 72°C for 1.5 min (extension), and a final extension at 72°C for 10 min.

### PCR product purification and sample pooling.

Ten microliters of each product was used to verify amplification by gel electrophoresis on a 2% agarose gel. Replicates yielding visible bands at bp 382 were pooled and purified according to provided protocol of the QIAquick PCR purification kit (Qiagen, Germany). Purified products were again quantified and quality checked at *A*_260_/*A*_280_ (NanoDrop spectrophotometer; Thermo Fisher Scientific, USA). Products were diluted to 10 ng/μl, and 5 μl of each sample was pooled for sequencing on a MiSeq sequencer (Illumina, USA).

### Sequencing and sequence processing.

Primers and sequence adapters were removed with the Illumina MiSeq Reporter (version 2.5). The sequences were further processed using scripts implemented in the R statistical computing environment with the DADA2 (version 1.10.1) package (27) (scripts are available on GitHub at https://github.com/lakarstens/ControllingContaminants16S). Briefly, sequences were quality filtered and trimmed (forward reads at 230 nucleotides [nt] and reverse reads to 210 nt) prior to inferring amplicon sequence variants (ASVs) with the DADA2 algorithm. ASVs, which group similar sequences together according to a model that considers sequence abundance and sequencing error, were chosen over traditional operational taxonomic units (OTUs) since they have a finer resolution (28–30). Chimeric sequences were removed with the approach implemented in the DADA2 package. Taxonomy was assigned for each ASV to the genus level using the RDP Naive Bayesian Classifier (31) implemented in DADA2 with the SILVA database (version 132). The R package phyloseq (version 1.26.1) (32) was used for storing the ASV table, taxonomy, and associated sample data and for calculating alpha-diversity measures. Expected values for alpha-diversity measures were calculated on the subset of the mock microbial community dilution samples that only contained expected sequences.

### Contaminant identification and removal.

Both negative-control filtering and relative abundance filtering were performed in R (version 3.5.2) using custom-made scripts available on GitHub (https://github.com/lakarstens/ControllingContaminants16S). Negative-control filtering was performed by removing any ASV that had a nonzero abundance in the mock community dilution series. Abundance filtering was performed transforming the mock microbial community data set to relative abundances and then setting any ASV values below the specified value (0.01, 0.1, and 1%) to zero.

Decontam (version 1.2.1) (20) was used to identify ASVs with significant inverse correlations with DNA concentration measured by a NanoDrop spectrophotometer (prior to library preparation; values provided in Table S1). We evaluated the performance of the Decontam frequency method using different user-defined classification threshold P* from 0.1 (default value) to 0.5. While values above 0.5 can be used, 0.5 is the threshold at which sequences will be classified as a contaminant if the contaminant model is a better fit than the noncontaminant model (20).

SourceTracker version 1.0.1 (21) was used to determine the amount of contamination in samples and recover the true microbial community of each sample. To test optimal inputs as source environments, we performed a series of analyses with the following samples labeled as source samples: contaminant profiles (created by removing the expected ASVs from the diluted mock microbial community samples), negative control (blank extraction controls that went through all the sample processing steps and microbial DNA-free water), and the expected mock microbial community profile (the undiluted mock microbial community sample).

### Software used.

R (version 3.5.2) (33) was used to process the raw sequencing data with the DADA2 package (version 1.10.1) (27). All analyses were completed in R (33) using the following packages: SourceTracker version 1.0.1 (21), Decontam version 1.2.1 (20), phyloseq version 1.26.1 (32), ggplot2 version 3.1.0 (34), tidyr 0.8.3 (35), and dplyr version 0.8.0.1 (36).

### Data availability.

The sequencing data supporting the conclusions of this article are available in the SRA under accession number SRP155048. The data and R markdown scripts necessary to reproduce the analysis presented in this paper are available at https://github.com/lakarstens/ControllingContaminants16S.
