# Supplementary figures and images for: Controlling for Contaminants in Low-Biomass 16S rRNA Gene Sequencing Experiments
Source: mSystems. 2019 Jun 4;4(4):e00290-19. doi: 10.1128/mSystems.00290-19 (PMC6550369; doi:10.1128/mSystems.00290-19)

## Filter method

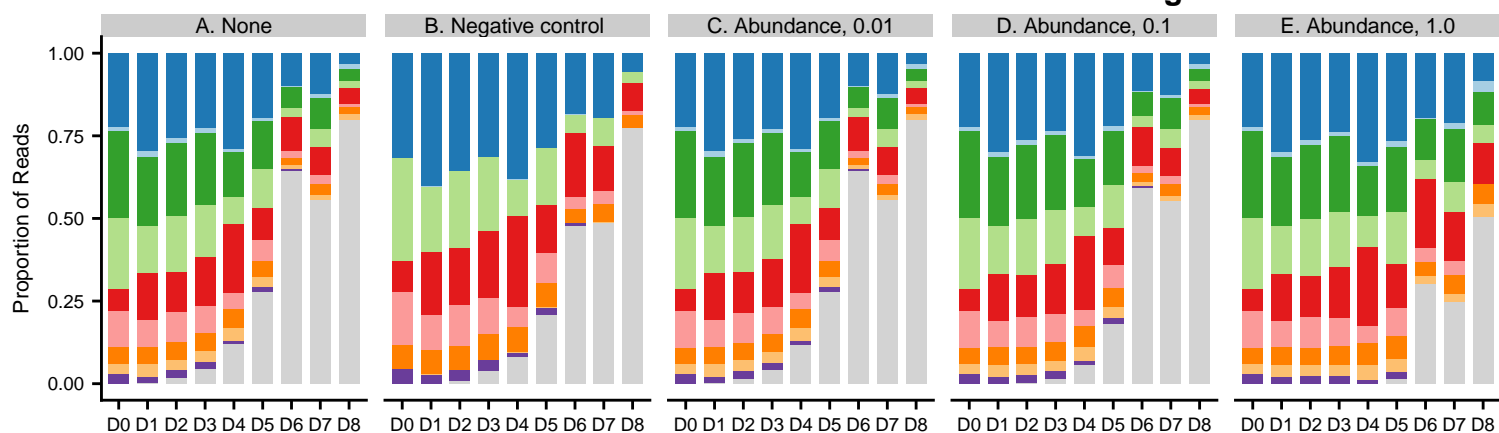

## Decontam frequency method

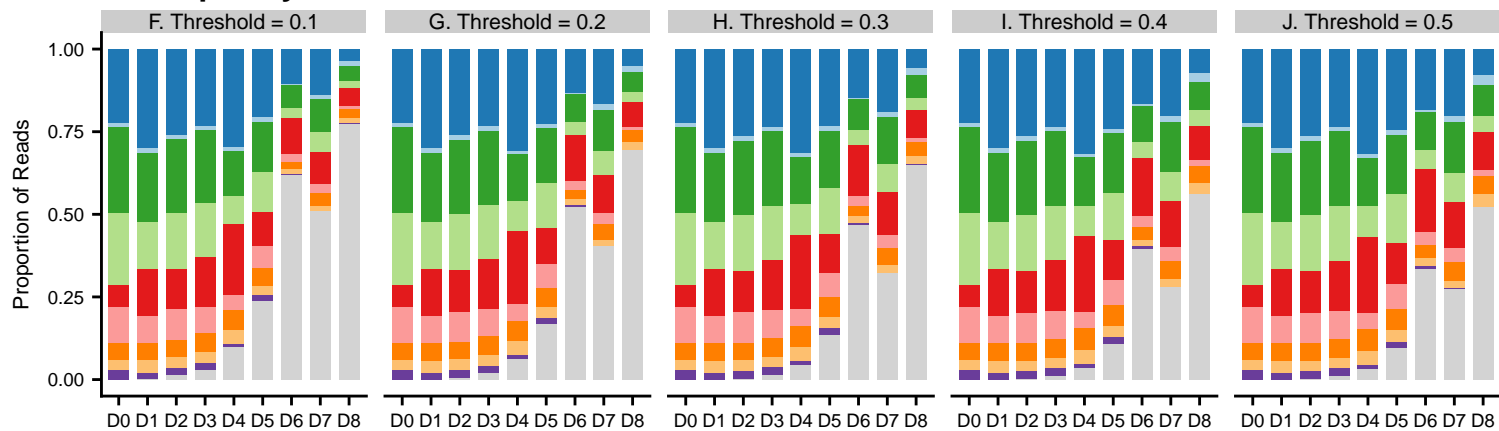

## SourceTracker method

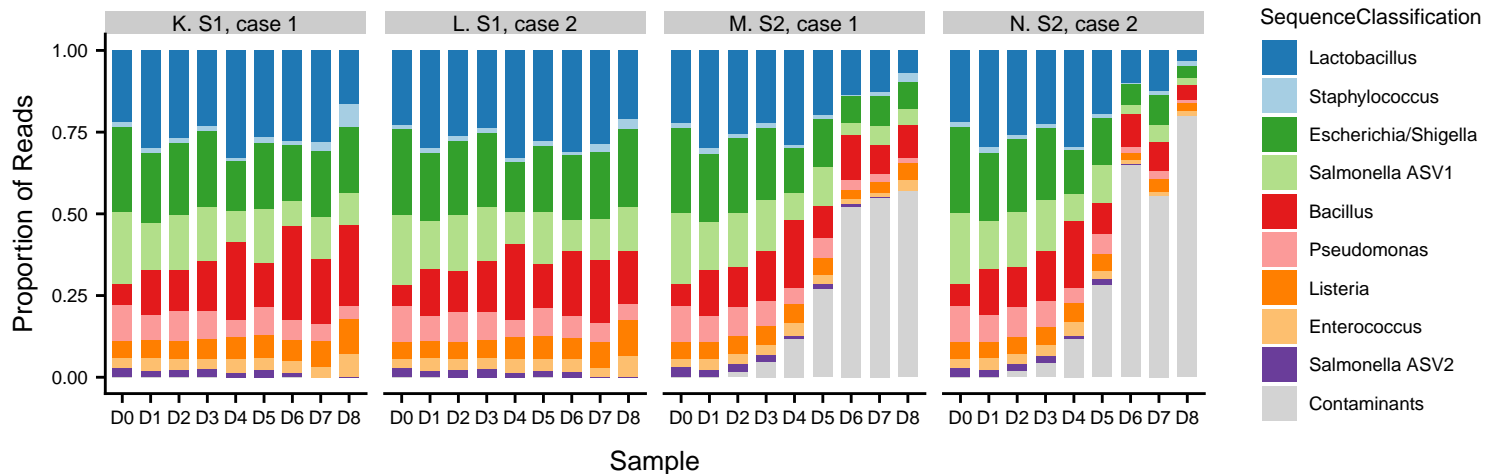

Supplement: FIG S1 [file mSystems.00290-19-sf001.pdf]
