# Supplementary material for: Controlling for Contaminants in Low-Biomass 16S rRNA Gene Sequencing Experiments
Source: mSystems. 2019 Jun 4;4(4):e00290-19. doi: 10.1128/mSystems.00290-19 (PMC6550369; doi:10.1128/mSystems.00290-19)
Supplement: FIG S2 [file mSystems.00290-19-sf002.pdf]

Figure S2

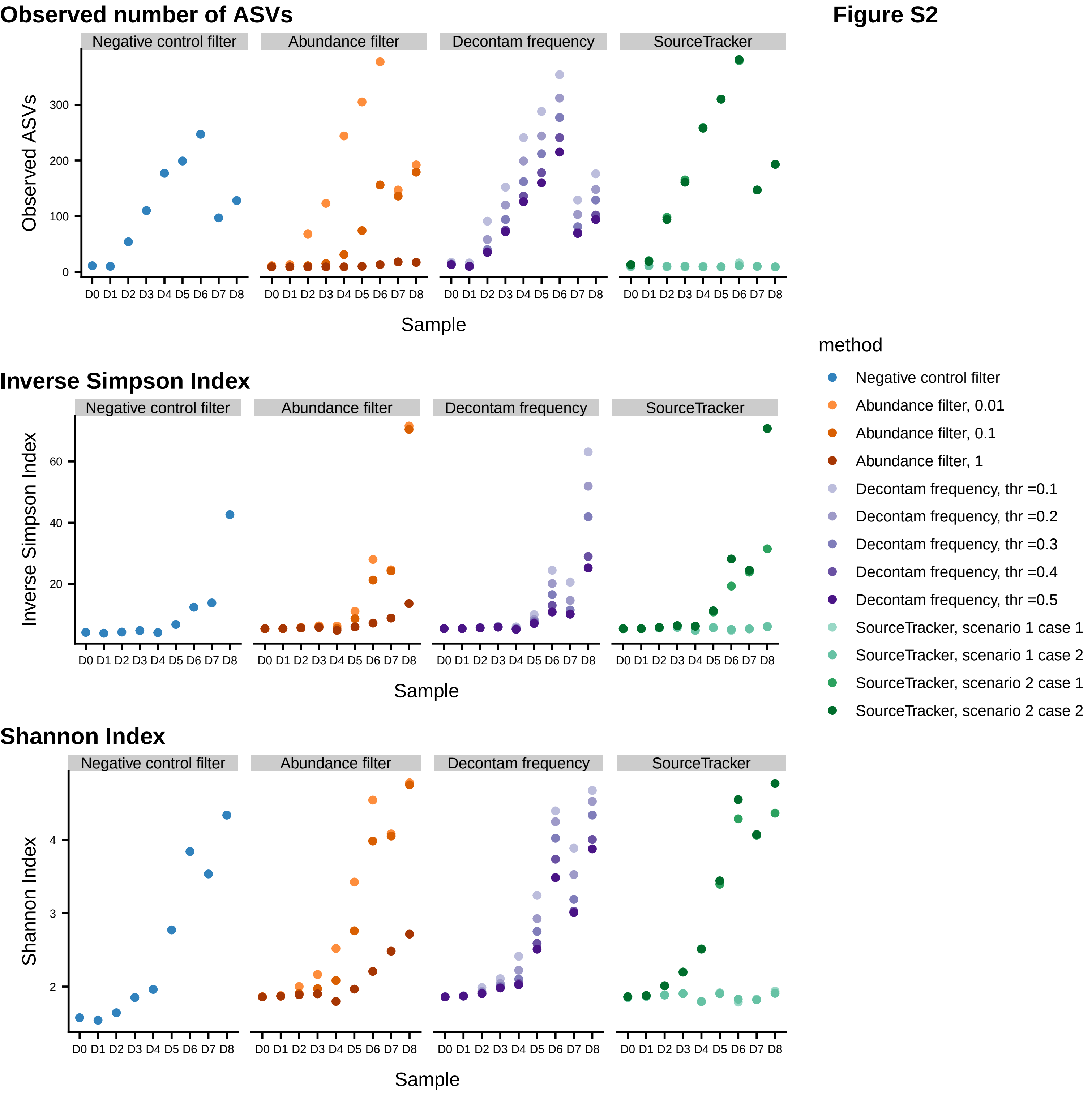
Observed number of ASVs
Negative control filter
Abundance filter
Decontam frequency
SourceTracker
Observed ASVs
300
200
100
0
D0 D1 D2 D3 D4 D5 D6 D7 D8
Sample
Inverse Simpson Index
Negative control filter
Abundance filter
Decontam frequency
SourceTracker
Inverse Simpson Index
60
40
20
D0 D1 D2 D3 D4 D5 D6 D7 D8
Sample
Shannon Index
Negative control filter
Abundance filter
Decontam frequency
SourceTracker
Shannon Index
4
3
2
D0 D1 D2 D3 D4 D5 D6 D7 D8
Sample
method
Negative control filter
Abundance filter, 0.01
Abundance filter, 0.1
Abundance filter, 1
Decontam frequency, thr =0.1
Decontam frequency, thr =0.2
Decontam frequency, thr =0.3
Decontam frequency, thr =0.4
Decontam frequency, thr =0.5
SourceTracker, scenario 1 case 1
SourceTracker, scenario 1 case 2
SourceTracker, scenario 2 case 1
SourceTracker, scenario 2 case 2
